# The Regulatory Subunit of PKA-I Remains Partially Structured and Undergoes β-Aggregation upon Thermal Denaturation

**DOI:** 10.1371/journal.pone.0017602

**Published:** 2011-03-04

**Authors:** Khanh K. Dao, Angel L. Pey, Anja Underhaug Gjerde, Knut Teigen, In-Ja L. Byeon, Stein O. Døskeland, Angela M. Gronenborn, Aurora Martinez

**Affiliations:** 1 Department of Biomedicine, University of Bergen, Bergen, Norway; 2 Facultad de Ciencias, Departamento de Quimica Fisica, Universidad de Granada, Granada, Spain; 3 Department of Structural Biology, University of Pittsburgh School of Medicine, Pittsburgh, Pennsylvania, United States of America; Institut Pasteur, France

## Abstract

**Background:**

The regulatory subunit (R) of cAMP-dependent protein kinase (PKA) is a modular flexible protein that responds with large conformational changes to the binding of the effector cAMP. Considering its highly dynamic nature, the protein is rather stable. We studied the thermal denaturation of full-length RIα and a truncated RIα(92-381) that contains the tandem cyclic nucleotide binding (CNB) domains A and B.

**Methodology/Principal Findings:**

As revealed by circular dichroism (CD) and differential scanning calorimetry, both RIα proteins contain significant residual structure in the heat-denatured state. As evidenced by CD, the predominantly α-helical spectrum at 25°C with double negative peaks at 209 and 222 nm changes to a spectrum with a single negative peak at 212–216 nm, characteristic of β-structure. A similar α→β transition occurs at higher temperature in the presence of cAMP. Thioflavin T fluorescence and atomic force microscopy studies support the notion that the structural transition is associated with cross-β-intermolecular aggregation and formation of non-fibrillar oligomers.

**Conclusions/Significance:**

Thermal denaturation of RIα leads to partial loss of native packing with exposure of aggregation-prone motifs, such as the B' helices in the phosphate-binding cassettes of both CNB domains. The topology of the β-sandwiches in these domains favors inter-molecular β-aggregation, which is suppressed in the ligand-bound states of RIα under physiological conditions. Moreover, our results reveal that the CNB domains persist as structural cores through heat-denaturation.

## Introduction

Adenosine cyclic 3′,5′-phosphate (cAMP) and guanosine cyclic 3′,5′-phosphate (cGMP) act as second messengers for many cellular processes [1]. Proteins that bind cyclic nucleotide monophosphate share ∼120-residue cyclic nucleotide binding (CNB) domains, often as repeats. The CNB fold – classified as the double-stranded beta-helix fold in SCOP (http://scop.mrc-lmb.cam.ac.uk/scop/) [2] − exhibits a β-sandwich topology and is present in several protein families, i.e. i) proteins with cAMP (or cGMP) binding domains, such as the regulatory subunit (R) of cAMP-dependent protein kinase (PKA), Rap1 guanine exchange factor (Epac), cGMP-dependent protein kinase (PKG), cNMP-gated ion channels and the gene activator protein (CAP) [3], and ii) proteins with cAMP-binding-like domains, such as the CO-sensing protein CooA [4] and the listeriolysin regulatory protein PrfA [5]. The sugar phosphate moiety of cAMP interacts with the so-called phosphate binding cassette (PBC), consisting of two pairs of antiparallel strands, linked by a short, one-turn helix that is characteristic of the double-stranded beta-helix fold. This short helix – referred to as B' in the CNB domain nomenclature (i.e. residues 200–205 in domain A (αB':A) and 324–329 in domain B (αB':B helices) for bovine RIα (see e.g. [3])) – is an important part of the PBC [6] and its structure is stabilized upon binding of cAMP [7], [8], [9].

All four isoforms of mammalian R (RIα, RIβ, RIIα, and RIIβ) include tandem A and B CNB domains. cAMP binding to both A and B sites in R induces conformational changes that lead to dissociation of the catalytic (C) subunit from the holoenzyme R2C2. In the holoenzyme, the cAMP affinity of R is markedly reduced [10]. Comparison of the crystal structures of RIα(103–376) with cAMP bound at the A and B sites [7] with that of RIα(91–379:R333K), complexed with C subunit [11], reveals large conformational changes that accompany the functional cycle of PKA. Recent NMR investigations of the binding of cAMP to the apo form of RIα(98–379) provided further novel information on the allosteric communication of cAMP binding in the regulatory subunit of PKA, with the αC:A and αC':A helices (residues 225–256) playing essential structural roles in A–B intersite communication [9].

The observed large conformational changes associated with regulation and function of PKA, elicited by cAMP or C subunit binding, require extensive structural plasticity in the RIα regulatory isoform, a property assumed to be a characteristic of other CNB-containing proteins as well [3], [12], [13]. Such plasticity could be brought about by extensive motion or flexibility, which often translates to low stability in other proteins, at least in the apo (ligand-free) states [14], [15]. For RIα, however, previous thermodynamic studies revealed it to be a thermodynamically rather stable protein, even more so in the presence of cAMP [16], [17].

Here we present biophysical investigations on the thermal denaturation of the RIα subunit with and without bound cAMP. The experimental investigations included circular dichroism (CD), differential scanning calorimetry (DSC), dynamic light scattering (DLS), thioflavin T (ThT) fluorescence and atomic force microscopy (AFM) experiments, and these experimental studies were complemented with molecular dynamics (MD) simulations. Both full-length RIα and the truncated RIα(92–381) form that contains the tandem CNB A and B domains are rather stable and this thermal stability is enhanced in the presence of cAMP. As evidenced through site specific mutants, binding of cAMP to the A domain is crucial for the enhanced stability. Importantly, the thermally denatured, full length and N-terminal truncated RIα proteins contain significant amounts of residual structure, and CD spectra exhibit characteristic β-structure features. ThT fluorescence indicates that the thermally denatured protein undergoes cross-β-aggregation and AFM reveals nonfibrillar, soluble oligomers after thermal denaturation. Finally, MD simulations and sequence analysis using the TANGO algorithm point to the B' helices in the phosphate-binding cassettes of both CNB domains as triggers of β-aggregation. Our results reveal that the CNB domains persist as relatively denaturation-resistant cores, a feature that might be advantageous for permitting large scale conformational changes that are of importance for the different functional states of RIα.

## Materials and Methods

### Mutagenesis, expression and purification of RIα proteins

DNA sequence corresponding to the human RIα(92–381), numbering according to Swiss-Prot Accession No. P10644, was amplified by PCR from the genomic DNA of human RIα [18] and cloned into the pGEX-2T vector with a Factor Xa-cleavable N-terminal maltose binding protein-tag (Pharmacia). Site directed mutagenesis of RIα(91–381) to create G201E-RIα(91–381) and G325D-RIα(91–381) was performed using the QuickChange^TM^ kit (Stratagene, La Jolla, CA).

Full length human RIα was purified using a pGEX-KG/RIα construct as reported [18]. Human RIα(92–381) was expressed in *E coli* BL21 (DE3) Codon Plus cells, induced at an OD_600_ of 0.6–0.7 with 1 mM IPTG, and grown for protein production for an additional 7–10 h at 30°C, when the bacteria were harvested. The pellets were resuspended in 100 ml of homogenization buffer (20 mM Na-phosphate, 150 mM NaCl, pH 7.3, 2 mM EDTA, 1% Triton X-100, containing 5 mM benzamidine, 1 mM DTT, 1 mM PMSF and 2 µg/µl leupeptin) and the bacteria broken by French press. The fusion protein was purified by affinity chromatography on amylose resin (New England BioLabs) with elution by 1 M methyl-α-D-glucopyranoside (Fluka, BioChemica) and cleaved by incubation overnight at 4°C with Factor Xa (New England BioLabs) (1∶300 protease:protein ratio), followed by gel-filtration chromatography on a HiLoad Superdex 200 HR (1.6 cm ×60 cm) (Pharmacia), and a second amylose resin column.

### Stripping off cAMP from the R subunits

Recombinant RI subunits (full-length and truncated RIα(92–381)) contain bound cAMP to various degrees of saturation after purification, and fully cAMP saturated R subunits can be prepared by adding cAMP (60 mM) prior to the last gel-filtration step. The cAMP-free proteins (apo-forms) were prepared as reported [9]. Briefly, the R subunits were incubated with 5 M urea in 10 mM K-phosphate, pH 7.4, 50 mM KCl, 1 mM EGTA for 5 h at 4°C, followed by filtration through a prepacked PD-10 column (GE Healthcare) and extensive buffer exchange using Amicon concentrators (Millipore, Billerica, MA). Refolding of the proteins was carried out by extensive dialysis against the same buffer without urea and purification on a HiLoad Superdex 200 HR (1.6×60-cm) column (Amersham Biosciences). The absence of cAMP in the protein solution was checked by measuring complete occupancy of cAMP sites by c[^3^H]-AMP (Amersham Biosciences) as reported [19].

### Circular dichroism (CD)

CD spectra were recorded on a Jasco J-810 spectropolarimeter, equipped with a peltier element for temperature control. Experiments were performed in 10 mM K-phosphate buffer, 50 mM KCl, pH 7.4 using 2 µM of cAMP-free (apo) RIα forms, in the absence or the presence of a large excess of cAMP (143 µM). Spectra were acquired at the indicated temperature over the 195–260 nm range at a scan rate of 50 nm/min. 4 scans were averaged for each spectrum. Buffer scans were recorded under the same conditions and subtracted. Thermal denaturation experiments were performed by monitoring changes in ellipticity at 222 nm at 1.5°C/min scan rate.

#### Differential scanning calorimetry (DSC)

Measurements were performed on a MicroCal VP-DSC differential scanning calorimeter (GE Healthcare) with a cell volume of 0.527 ml at the indicated scan rate, customarily 1.5°C/min. Scans were performed over the 15–95°C range, unless indicated. Samples of RIα(92–381) in its apo form (4–8 µM) were prepared in 10 mM K-phosphate buffer pH 7.4, 50 mM KCl, and centrifuged prior to measurements. cAMP was added at the indicated concentrations. Buffer-buffer baselines were acquired prior to the experiments and subtracted.

Chemical baselines were removed prior to data fitting, using a cubic baseline routine (manufacturer's software). The subsequent excess molar heat capacity profiles (Cp(ex)) were fitted to the following equation:
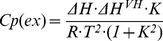
(1)where *ΔH* is the calorimetric enthalpy, Δ*H* ^VH^ is the van't Hoff enthalpy, *R* is the ideal gas constant, *T* is the absolute temperature (in K) and *K* is the equilibrium constant:
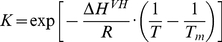
(2)with *T*_m_ as the transition temperature.

The theoretical Δ*H* (Δ*H*_calc_) based on crystal structures were obtained using well-known structure-energetics relationships [20], [21] as described previously [22].

### Thioflavin T (ThT) fluorescence

A Cary Eclipse fluorescence spectrophotometer equipped with a temperature-controlled Peltier multicell holder (Varian) was used for monitoring ThT binding by fluorescence. The excitation wavelength was 440 nm and emission wavelength 482 nm. A stock solution of 1 mM ThT (Sigma) was prepared in SuperQ Millipore water and stored at 4°C and protected from light until used. The protein solution was prepared in 10 mM K-phosphate buffer, 50 mM KCl, pH 7.5 containing 60 µM ThT, immediately before the measurement. The final protein concentration was 2–10 µM. The ThT dye was shown not to bind to the native monomeric RIα(92–381) or dimeric RIα. Temperature dependent fluorescence measurements were performed at the indicated scan-rate, while kinetic measurements were monitored at the indicated temperature.

### Atomic force microscopy (AFM) imaging

Samples were prepared by spreading 10 µL of RIα(92–381) (8 µM protein concentration) on a 25×25 mm freshly cleaved mica surface, incubated for 5 min and gently washed in milliQ water. When dried, the sheets were transferred directly to the AFM instrument for imaging. AFM imaging was carried out in air at room temperature using the tapping mode (AC mode) on an MFP-3D-Bio^TM^ atomic force microscope (Asylum research, Santa Barbara, CA). Silicon cantilevers, ACL, from AppNano with a typical spring constant of 48 N/m were used. Images were captured with a resolution of 256×256 pixels and the scan rate was adjusted for each sample to a value between 0.5 and 1 Hz. At least three regions of the sample surface were investigated to confirm homogeneity. All images were processed by plane fitting using IGOR PRO (Wavemetrics, OR).

### Dynamic light scattering (DLS) measurements

Protein molecular masses at 22 and 60°C were estimated using a DynaPro LSR (Protein Solutions Inc. USA) DLS instrument with Temperature Controlled Micro Sampler. 25 data acquisitions were collected for each independently protein sample in 10 mM K-phosphate buffer, 50 mM KCl, pH 7.5. The data were analyzed using Dynamics V6 (Protein Solutions) to obtain the hydrodynamic radius (*R*_H_), given as the mean size of the dominant peak.

### Molecular dynamics (MD) simulations

MD simulations were performed using Amber9 [23], applying the Amber ff99SB [24] and gaff [25] force fields. The electrostatic surface potential of cAMP was calculated at the HF 6–31G* level and atomic point charges were assigned by a two-step RESP charge fitting procedure [26] with Antechamber [27]. Coordinates for the truncated regulatory subunit RIα(113–376) in complex with cAMP were taken from the 2.8 Å resolution crystal structure of Su et al. [7] (PDB code 1RGS). All crystallographic water molecules were removed. The initial model of the apo form of R1α(113–376) was prepared by removing the coordinates of cAMP from the coordinate file. TIP3P [28] water molecules were added to create a truncated octahedron, with a minimum of a 10 Å water layer between the octahedron edges and the nearest solute atoms. Both systems were energy minimized and then heated to 300 K over 50 ps with weak harmonic restraints on the solute at constant volume. Another 100 ps equilibration of the system was then performed at constant pressure. The simulations were performed in the presence and absence of cAMP, without positional restraints, initially at 300 K for 100 ns at constant volume (NVE ensemble). Use of SHAKE constraints [29] on bonds involving hydrogen atoms allowed a 2 fs time step. The temperature was increased gradually to 450 K over 15 ns, applying a 1fs time step at constant volume with Langevin temperature regulation (collision frequency of 1/ps). The structures were immediately cooled down again to 300 K over 15 ns. Further equilibration at constant pressure with Langevin temperature regulation was performed for 20 ns at 300 K after the temperature jump. Electrostatic forces were computed using Particle Mesh Ewald summation [30] and snapshots for subsequent analysis were taken every 1000 dynamics steps of the simulations.

### TANGO algorithm

Regions involved in beta aggregation were predicted using the TANGO algorithm [31].

## Results

### Secondary structure and stability of RIα investigated by circular dichroism (CD). Effect of cAMP

**Figure 1:**
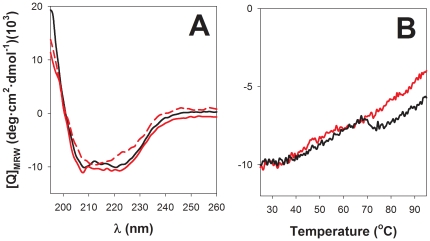
Thermal unfolding of full-length RIα monitored by CD spectroscopy. (A) Far-UV CD spectra at 25°C of apo-RIα, stripped of endogenously bound cAMP (red solid line), RIα-cAMP, with excess (143 µM) cAMP (black line), and of apo-RIα at 75°C (red dashed line). The CD spectrum taken at 25°C after heating apo-RIα to 95°C is similar to that shown by the red dashed line. (B) Temperature dependence of the ellipticity at 222 nm at 1.5°C/min scan rate, for apo-RIα (red) and cAMP saturated RIα (black). Further details are provided in the Experimental section. MRW, mean molar residual ellipticity. All experiments were performed with 2 µM full-length RIα protein.

**Figure 2:**
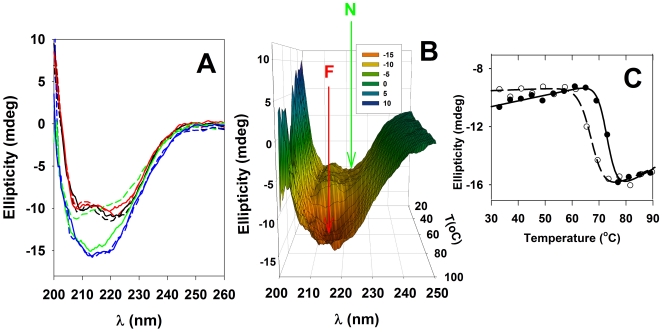
Thermal denaturation of RIα(92–381) monitored by far-UV CD. A) Selected far-UV CD spectra of apo (solid line) and cAMP-saturated (10 fold-molar excess of cAMP; dashed line) RIα(92–381), at 25°C (black), 45°C (red), 69°C (green) and 85°C (blue). B) CD spectra of apo-RIα(92–381) were recorded every 4°C over the temperature interval 25–95°C at a scan rate of 2°C/min, with 4 scans at each temperature (100 nm/min). N indicates the native and F the final (irreversible) states. C) Temperature dependence of CD signal at 216 nm in the apo (○) and cAMP-saturated states (•). All experiments were performed at a protein concentration of 2 µM.

The far UV-CD spectrum of dimeric full-length human RIα, both in the apo- or cAMP-bound forms, exhibit minima at 209 and 222 nm (Figure 1A; see also [16], [17]), characteristic of proteins that contain significant amounts of α-helix. Monitoring the ellipticity at 222 nm as a function of temperature from 25 to 95°C failed to reveal a clear cooperative unfolding transition (Figure 1B). Instead, an unusual and pronounced dip in the temperature-dependent CD profile occurs at 70–72°C for RIα incubated with excess cAMP, and a less pronounced dip occurs at about 10°C lower temperature for apo-RIα (Figure 1B). At >75°C, the CD spectra (ellipticity minima at ∼212 nm) revealed a large amount of residual β-structure both in the absence and presence of cAMP (see Figure 1A for the spectrum of apo-RIα). For RIα heated to 95°C and cooled back down to 25°C, the CD spectrum with the 212 nm minimum was maintained (Figure 1A), indicating that an irreversible structural conversion with a change in secondary structure (from α-helix to β-sheet) and/or aggregation (see below) had occurred. The truncated monomeric RIα(92–381) protein that contains the tandem CNB A and B domains, but lacks the highly helical N-terminal docking/dimerization (D/D) domain [32], was selected to further investigate determinants for thermal stability of RIα and the apparent structural conversion upon denaturation. Functionally, RIα(92–381) exhibits similar affinity as full-length RIα both for cAMP and the catalytic C subunit [10], [33]. At low temperatures, the far-UV CD spectrum of RIα(92–381) resembled that of RIα, whether recorded in the absence or presence of cAMP, but the ellipticity values after denaturation at high temperatures were markedly more negative for the truncated protein relative to full-length protein (Figure 2A). Moreover, the increased negative ellipticity, with the 212–216 nm minimum, was maintained when RIα(92–381) was cooled down to 25°C (Figure 2A), indicating that the N-terminal D/D domain and dimerization of full length RIα did not contribute to the observed irreversible thermal transition. We then performed a detailed temperature-dependent CD analysis of the truncated form (Figure 2B), which also yielded midpoint transition temperatures of ∼67–68°C and ∼73–74°C for apo- and cAMP-bound proteins, respectively (shown in Figure 2C). The equilibrium [^3^H]cAMP binding ability of the truncated form, measured at 25°C by the ammonium sulfate precipitation method as described [9], was also found to be irreversibly lost after heating the samples at temperatures ≥68°C for 5 min, and subsequent cooling on ice for 10 min (data not shown).

By using the K2D algorithm [34] for analysis of the CD spectra for cAMP-saturated RIα(92–381) we estimated that the α-helix and β-strand content in the native protein is 33% and 16%, respectively. These values changed to 24% (α-helix) and 36% (β-strand) after heating to 95°C. The crystal structure of a similar cAMP-saturated mutant [7] exhibits 31% α-helix and 25% β-sheet, suggesting that the CD/K2D method provides a good estimate for the α-helix content in the native protein while a poorer agreement was found for β-structure. Nevertheless, these results indicate that the β-structure increases substantially upon thermal denaturation, with a small decrease in α-helix. Similar changes in the CD spectra involving α→β transitions have been seen for proteins that go from a soluble monomeric state to a cross-β-aggregated state and for which the β-structure is stabilized by intermolecular interactions [35], [36]. In fact aggregation without conversion of α-helix to β-sheet may also result in β-like CD spectra [37] and therefore we performed several further analyses to test whether the change also involves aggregation for RIα(92-381)'s α→β transition.

### Differential scanning calorimetry (DSC)

**Figure 3:**
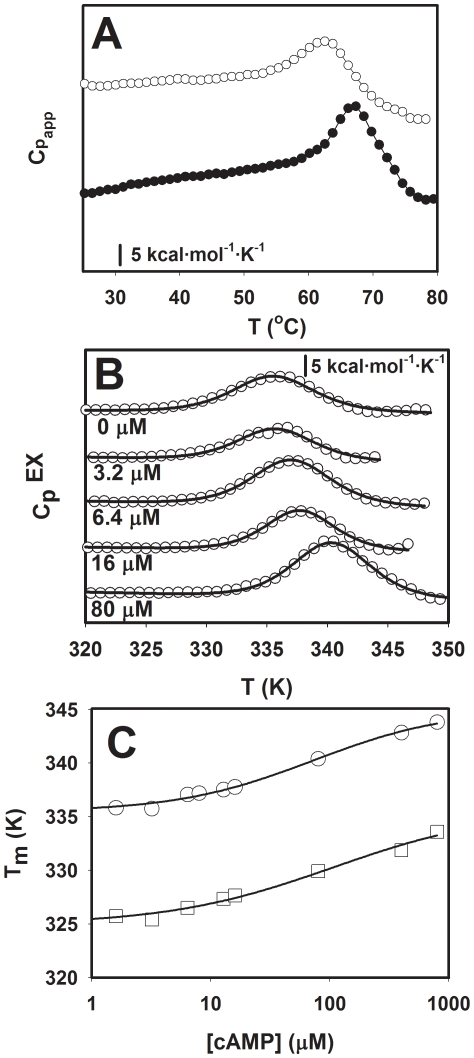
DSC analysis of RIα(92–381). A) Profiles of apparent molar heat capacities (*C*p_app_) *vs* temperature (*T*) for RIα(92–381), in the apo state (○) and in the presence of a 10 fold molar excess of cAMP (•). B) Profiles of excess molar heat capacity (*C*p_EX_) after subtraction of the corresponding baselines for increasing cAMP concentrations. C) Dependence of the T_m_ value on cAMP concentration for RIα(92–381) (○) and G325D-RIα (92–381) (□). The apo-forms of both proteins were used for the titrations. Note that the scale is logarithmic and the lines are only to guide the eye.

Characterization of the thermal response of RIα(92–381) by DSC reveals a single unfolding transition with midpoint melting temperature (*T*_m_) of 62.5°C for the apo protein, which is up-shifted in the presence of cAMP (Figure 3). Interestingly, the T_m_ obtained by DSC is about 5°C lower than the mid point temperature for the structural transition measured by CD (Figure 2), but equally irreversible. Therefore, a full equilibrium thermodynamic analysis is not applicable. However, the kinetic distortion is not large whether cAMP is present or not, as judged by the symmetry of the transitions (Figure 3A,B) and a close to unity Δ*H*/Δ*H* ^VH^ ratio (0.8–1.2) at all concentrations of cAMP tested. Furthermore, the scan-rate dependence of *T*_m_ within the 0.3–1.5 K/min range was negligible (data not shown), supporting that at these scan-rates it may be permissible to use equilibrium thermodynamics analysis [22], [38]. Such analysis yields *T*_m_ = 62.5±0.1°C, Δ*H* = 111.6±2.9 kcal/mol and Δ*H* ^VH^ = 96.3±2.1 kcal/mol for apo-RIα(92–381) and *T*_m_ = 71.0±0.1°C, Δ*H* = 120.4±1.2 kcal/mol and Δ*H* ^VH^ = 139.9±1.4 kcal/mol for the cAMP-saturated protein.

The G325D-RIα(92–381) mutant has silent cAMP binding site B, but retains the ability to inhibit the C subunit of PKA, acting as a dominant negative PKA inhibitor in intact cells [10]. This mutant has decreased thermal stability in its apo-form (*T*_m_ = 52.1±0.1°C) compared to wild-type (wt) RIα(92–381) (*T*_m_ = 62.5±0.1°C) (Figure 3C), indicating that the native conformation of site B contributes to the stability of RIα even when site B is non-occupied. On the other hand, G325D-RIα(92–381) shows a similar ∼10°C increase of *T*_m_ upon cAMP binding as wt RIα(92–381). Thus, binding to site A alone is sufficient to provide the same increase in thermal stability as caused by cAMP binding to both cAMP binding sites. Therefore, the CNB site A, that also is formed by some residues from domain B, such as Trp262 [7], [9], is an important determinant for allowing ligand-induced enhancement of stability to occur. The significance of the CNB site A for the stability of the regulatory subunit is further supported by the fact that the silent A site mutant G201E-RIα(92–381) is too unstable for detailed denaturation studies.

### Structure-energetics relationships

A theoretical Δ*H* (Δ*H*_calc_) value at the denaturation temperatures was calculated based on the crystal structure of the cAMP saturated protein (PDB 1RGS; [7]), using the structure-energetics relationships developed by Freire and co-workers [20], [21] (see [22] for specific equations). The Δ*H*_calc_-value of 236 kcal/mol (Table 1) lies within the range expected for a protein of this size. Calculations based on other crystal structures of the truncated apo-protein also result in larger values than the experimental Δ*H*, i.e. PDB 1RL3 [39] with cAMP absent in site B and cGMP in site A yields Δ*H*_calc_ = 212 kcal/mol, and PDB 2QCS [11] bound to the catalytic C subunit yields Δ*H*_calc_ = 230 kcal/mol (Table 1), compared to the experimentally determined Δ*H* value for RIα(92–381) of 111–120 kcal/mol. One reason for a large difference between the theoretically predicted and experimentally determined Δ*H* values may pertain to substantial residual structure in the thermally denatured state [40].

**Table 1:** Structural-derived energetic parameters calculated based on the crystal structures of N-terminal truncated forms of RIα.

Parameter	cAMP-boundPDB 1RGS(residues 113–376)	cAMP-free (apo)*PDB 1RL3(residues 109–376)	cAMP-free (apo)** PDB 2QCS(residues 90–380)
Δ*ASA*_ap_ (Å^2^)	18746	19535	19938
Δ*ASA*_pol_ (Å^2^)	10598	11526	12194
Δ*C*_p_calc_ (kcal/K·mol)	5.7	5.8	5.8
Δ*H*_calc*_*60_ (kcal/mol)	174.5	197.0	214.6
*T*_m_ (°C)	71	62.5	62.5
Δ*H*_calc_at *T*m_ (kcal/mol)	236	212	230

Experimental values for cAMP-free (apo)-RIα(92–381); *T*_m_ = 62.5±0.1°C, Δ*H* = 111.6±2.9 kcal/mol and Δ*H* ^VH^ = 96.3±2.1 kcal/mol.

Experimental values for cAMP-bound RIα(92–381); *T*_m_ = 71.0±0.1°C, Δ*H* = 120.4±1.2 kcal/mol and Δ*H* ^VH^ = 139.9±1.4 kcal/mol (with 800 µM cAMP; 4 µM RIα(92–381)).

*The cGMP present in site A was not included the calculations.

**The C structure was not included in the calculations.

Theoretical values for the unfolding heat capacity (Δ*C*_p_calc_) and enthalpy changes at 60°C (Δ*H*_calc_60_) and at the T_m_ (Δ*H*_calc_at Tm_) were calculated from the changes in apolar and polar accessible surface area (Δ*ASA*_ap_ and Δ*ASA*_pol_) upon unfolding using structure-energetics correlations (see [22] for equations).

In conclusion, the combined CD, DSC and theoretical structure-energetics calculations indicate that the low experimental Δ*H* may arise from a partial unfolding of the protein prior to the irreversible structural change associated with an apparent α→β transition.

### Aggregation of RIα(92–381); ThT fluorescence, atomic force microscopy (AFM) and dynamic light scattering (DLS)

**Figure 4:**
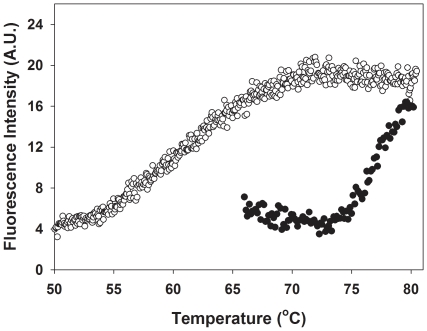
Thioflavin T (ThT) binding. ThT fluorescence intensity was monitored at 482 nm, with excitation at 440 nm in the presence of 8 µM apo-RIα(92–381) without (blue symbols) and with 143 µM cAMP (red symbols), 60 µM ThT, at pH 7.5. The scan rate was 1.5°C/min. Control experiments showed no increase in fluorescence intensity at 482 nm when the sample was maintained at 20°C for 2 h.

As mentioned above, the increased negative ellipticity at 216 nm is indicative of increased β-structure, either by intra-subunit formation of β-sheets or β-turns and/or formation of extended structures formed by aggregation via inter-chain cross-β interactions [35], [36], [41]. We further investigated if cross-β aggregation was occurring by monitoring ThT fluorescence. Thermal denaturation of RIα or RIα(92–381) at neutral pH and concentrations of 2–20 µM protein yielded only soluble aggregates and did not lead to macroscopic precipitation, not even after storage of the denatured proteins at room temperature for at least one week. However, binding of ThT was clearly observed (Figure 4) and the increase in ThT fluorescence occurred at the temperatures where the α→β conversion was noted in the CD spectrum (Figure 2). In general, ThT fluorescence is used to monitor fiber/fibril formation in amyloids [42], [43], [44], although nonfibrillar, soluble oligomers that contain β structure can also be detected by ThT fluorescence [41], [45].

**Figure 5:**
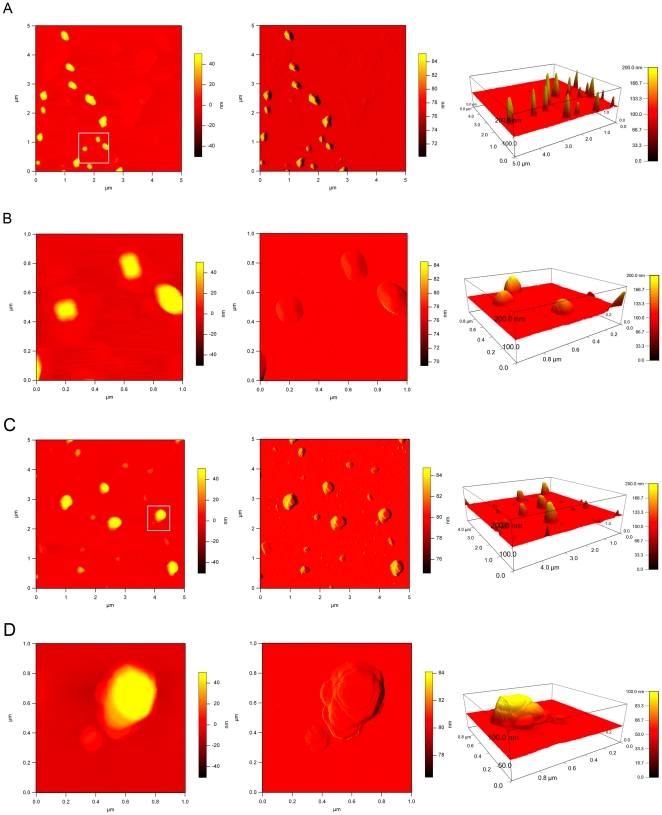
AFM analysis of the RIα(92–381) aggregates. The left column displays the height image, the middle column shows the amplitude image and the right column represents the height image in 3D. A and B were scanned right after thermal denaturation of RIα(92–381), while C and D were scanned after one week incubation of the denatured protein at room temperature. The scan in B and D is a close-up of the area marked with a white rectangle in A and C, respectively.

Further characterization of the size and shape of the aggregates was carried out using AFM since structures involving cross-β type of aggregation are readily observed by this technique [46]. In the present case quite regular aggregates were seen for the thermally denatured RIα(92–381) (Figure 5A,B) with lateral dimensions ranging from ∼100 to ∼200 nm and heights of 50 to ∼200 nm. Based on DLS, the aggregates exhibit a hydrodynamic radius of ∼112±5 nm, corresponding to an estimated molecular mass of ∼200 kDa, and the low polydispersity of the thermally denatured sample is indicative of homogeneous aggregate formation, without signs of further growth. However, incubation of the denatured protein aggregates at room temperature results in further aggregation into larger species (Figure 5C,D). These larger assemblies look disc-like, with distinct ridges. A typical particle contains a total of ∼8 ridges (as seen in Figure 5D), with smaller assemblies exhibiting only two ridges.

### TANGO and molecular dynamics (MD) simulation of thermal denaturation

**Figure 6:**
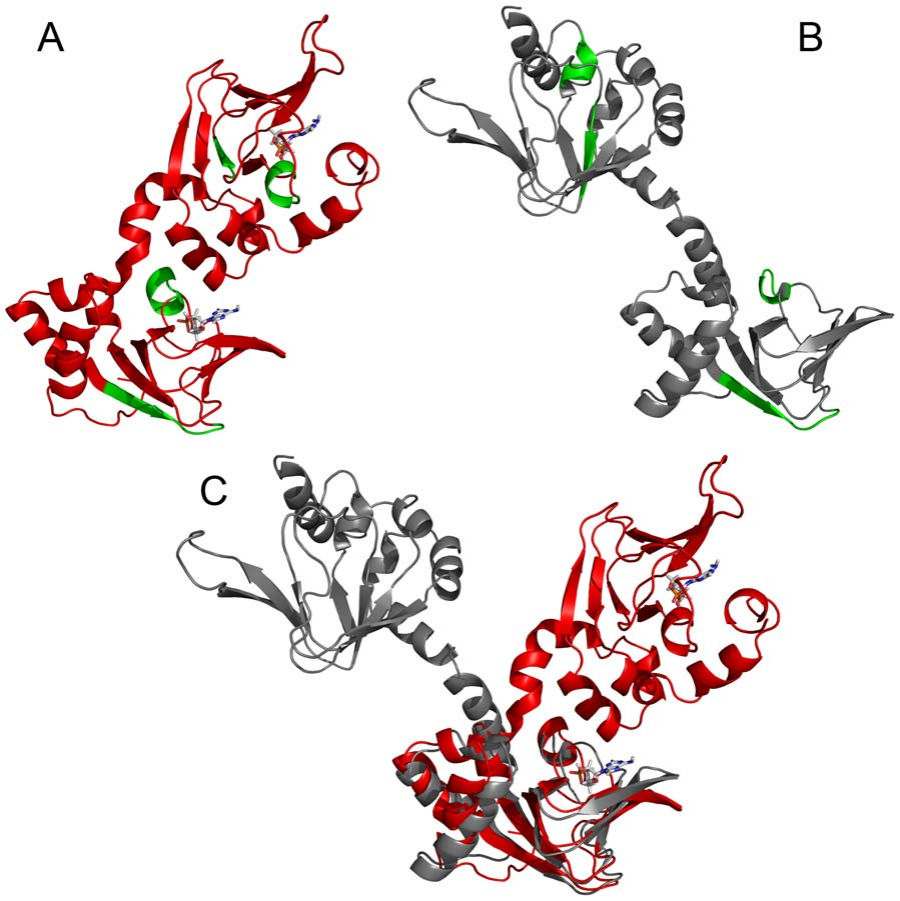
Aggregation prone areas as indicated by TANGO and high temperature MD. A) Structural mapping of the β-type aggregation motifs implicated by the TANGO algorithm onto the structure of bovine RIα(113–376) (PDB 1RSG) [7], regions 153–159, 201–206, 290–294 and 325–329 are shown in green. 201–206 and 325–329 constitute the B' helices of CNB domains A and B, i.e. αB':A and αB':B, respectively. B) Structure after the MD simulation at 450 K (cAMP was deleted from the complex (PDB 1RSG) at the beginning of the MD simulation; see text for details). (C) Superposition of the initial cAMP-free structure (red) and after high-temperature MD simulation (gray). The superposition was carried out by best-fitting of CNB domain A. RMSD values (C-alpha atoms) are 3.9 Å for the CNB A domains and 15.7 Å for the entire protein. Note that the change in relative orientation of the domains around the interdomain C/C' helices occurs without any significant changes in the structure of the domains β-sandwich.

The TANGO algorithm has been developed to identify sequences involved in the aggregation of proteins [31]. For RIα(92–381), TANGO points to regions encompassing residues 153–159, 201–206, 290–294 and 325–329 (numbering according to the bovine sequence) as being most prone to aggregate in a β-type of interaction (see green motifs in Figure 6A). Two of these regions reside in the β-strands of the CNBs A and B β-sandwich, while 201–206 and 325–329 correspond to the short B' helices that form part of the PBC in the CNB domains A and B (referred to as αB':A and αB':B, respectively). The B' helices are important functional and structural motifs that stabilize cAMP binding by an N-terminal capping mechanism with the phosphate group [7], [8], and we have recently shown that these helices are structured only in the cAMP-bound and not in the ligand-free protein [9].

We complemented all the above experimental investigations on the thermal denaturation of RIα(92–381) by MD simulations at high temperature (450 K). Such simulations have been successfully used to “accelerate” putative conformational changes over high activation barriers [47] or to investigate the thermal unfolding of proteins [48]. For thermal unfolding simulations, the timescale for the conformational changes is compressed, without affecting the pathway of unfolding [48]. MD simulations were performed using the RIα structure both with and without bound cAMP (PDB ID 1RGS [7], using a simplified ligand-free model in which the ligand coordinates were deleted from the complex. Simulations were carried out at 300 K for 100 ns and the temperature was progressively increased to 450 K over 15 ns, prior to cooling down to 300 K over 15 ns and further equilibration for 20 ns at this temperature. No conformational changes from the initial crystal structure were observed for the cAMP bound protein (data not shown). However, the high temperature MD simulations of the apo-protein resulted in a melting of the interdomain αC:A helix, with concomitant changes in orientation of the two CNB domains (Figures 6A–C). Interestingly, the B' helices that were implicated by TANGO as potential aggregation regions were also partially unfolded and became more solvent exposed at the end of the MD simulation (Figures 6A,B).

## Discussion

### The thermal denaturation of RIα; stability, flexibility and a highly structured denatured state

The activity of RIα depends on substantial flexibility and plasticity of the protein, given its need to adopt very different conformations along its functional cycle. Based on the available crystal structures, the protein changes from a compact structure when bound to cAMP [7], to a more extended conformation when bounding and inhibiting the C subunit of PKA [11] (see [3], [49] for a review). Despite its high plasticity, the RIα protein is thermodynamically quite stable, notably when saturated by cAMP. Whereas chemical denaturation by chaotropes such as urea and guanidinium chloride is fully reversible [16], [17], [50], thermal denaturation is irreversible [51] (and this work). In that regard RIα is not unusual, since many proteins unfold and refold by different mechanisms and populate different intermediate unfolding states depending on the denaturing agent (chaotropes, pH or temperature) [52], [53], [54], [55]. Moreover, aggregation at high temperatures is known to impede reversibility [56]. As described here, RIα's irreversible thermal denaturation does not involve global protein unfolding; only partial unfolding occurs, exposing hydrophobic regions which subsequently aggregate.

The experimental Δ*H* obtained from DSC for RIα(92–381), i.e. 120.4±1.2 kcal/mol for the cAMP saturated protein, indicates that a significant amount of ordered structure is lost upon thermal denaturation. Nevertheless, the measured experimental Δ*H* value is much lower than the theoretically calculated Δ*H*_calc_ (236 kcal/mol), estimated based on total unfolding of the protein structure (Table 1). This discrepancy supports the notion that a large amount of residual structure remains in the thermally denatured state, as also indicated by the CD results.

### Aggregation of RIα

ThT fluorescence has customarily been used to characterize amyloid fibril formation of proteins. However, ThT also binds to non-fibrillar soluble aggregates with β structure [41], [45]. The latter state is also present in RIα. AFM of the denatured protein, as well as DLS measurements at high temperature, both point to a non-fibrillar oligomeric structure of the RIα(92–381) aggregates. As to the nature of the β-strand association we can only speculate. One mechanism of β-strand association is the capture of unstructured segments into β-sheets [57], and loops and short helices that link β-strands have also been proposed to act in aggregation of proteins [58], [59]. The short αB':A and αB':B helices, located at the C-terminus of the edge strand in the PBC domains' β-sandwich (Figure 6A,B) are good candidates for inter-molecular association in RIα, notably in the absence of cAMP. They are also implicated by TANGO as motifs with high propensity for β-aggregation. Loosening of the protein structure that accompanies increased thermal fluctuations at higher temperatures exposes these short helices and the other aggregation-prone regions (Figure 6B), thereby facilitating inter-molecular associations and formation of the oligomeric structures (Figure 5A). In the native RIα structure the aggregation-prone areas are protected; residues 153–159 in CNB A are covered by CNB B, the αB':A helix (201–206) is protected by the interdomain αC:A helix, and the areas around 290–294 and the αB':B helix (325–329) are covered by the C terminal αC:B helix. Such protection strategies for avoiding β-aggregation have been recognized in other proteins with high β-sheet content, notably for beta-helix fold ones [60].

Amyloidogenic proteins and peptides generally exhibit substantial conformational plasticity and have been found to engage multiple aggregation pathways, depending on environmental conditions. As a result, a variety of aggregated oligomeric structures can be formed that lead to amorphous aggregates or fibrils [46]. Under appropriate destabilizing conditions, even proteins not related to any kind of amyloidogenic disease can form fibrils [61], [62], [63]. Pre-fibrillar precursors are often formed from initial oligomeric assemblies and amyloid formation from oligomers and pre-fibril assemblies has been extensively studied *in vitro* [36], [64]. Therefore, for each protein, extensive detailed condition-dependent investigations are necessary to assess this protein's individual behavior. Such studies of RIα aggregation were carried out here. No fibril-type aggregation of RIα was however observed, although soluble aggregates clearly were formed. Nevertheless, we would like to stress that the thermally induced β-type aggregation of RIα is not expected to occur *in vivo*, where in the PKA functional cycle RIα is either bound to cAMP or to the C subunit [11], [13]. Moreover, a large proportion of PKA is anchored to discrete cellular membranes by the A-kinase anchor proteins (AKAPs) [65], and it cannot be excluded that this anchoring also can contribute to stabilize the RIα protein, possibly preventing inter-molecular contacts and aggregation. Nevertheless, it may be valuable to examine the possibility whether aggregation could take place in some variants of RIα, associated with the Carney complex [66]. This disease exhibits large phenotypic heterogeneity and while about half of the mutations are associated with nonsense-mediated mRNA decay, leading to RIα haploinsuficiency, it is increasingly evident that altered protein variants may also contribute to the pathology [67], [68].

In conclusion, our results indicate that thermal denaturation of RIα involves a partial loss of native packing (native state → partially unfolded), as suggested by DSC and MD simulations, with subsequent self-association via β-type aggregation (partially unfolded → cross-β-intermolecular aggregates → disc-like soluble aggregates), as indicated by CD, ThT fluorescence and AFM experiments. It seems highly probable that the B' helices in the CNB domains constitute the structural regions that trigger β-aggregation, while the β-sandwich of the domains forms relatively denaturation-resistant cores. These cores are the stable structural units of RIα that persist throughout the large conformational changes necessary for function and regulation.
